# Altered Intrinsic Regional Spontaneous Brain Activity in Patients With Severe Obesity and Meibomian Gland Dysfunction: A Resting-State Functional Magnetic Resonance Imaging Study

**DOI:** 10.3389/fnhum.2022.879513

**Published:** 2022-05-19

**Authors:** Yi Liu, Sheng-Xing Tan, Yu-Kang Wu, Yan-Kun Shen, Li-Juan Zhang, Min Kang, Ping Ying, Yi-Cong Pan, Hui-Ye Shu, Yi Shao

**Affiliations:** ^1^Department of Gastrointestinal Surgery, The First Affiliated Hospital of Nanchang University, Nanchang, China; ^2^Department of Ophthalmology, The First Affiliated Hospital of Nanchang University, Jiangxi Branch of National Clinical Research Center for Ocular Disease, Nanchang, China

**Keywords:** severe obesity, meibomian gland dysfunction, regional homogeneity, resting state, functional magnetic resonance imaging, Hospital Anxiety Depression Scale

## Abstract

**Purpose:**

To evaluate potential regional homogeneity (ReHo) cerebrum function lesions in people with severe obesity and meibomian gland dysfunction (SM) and probe the connection between aberrant cerebrum activity and clinical manifestations.

**Patients and Methods:**

An aggregation of 12 patients with SM, and 12 healthy controls (HCs) closely matched in age and gender were enrolled. We applied corneal confocal microscopy and fundus angiography to compare imaging distinctions between the two groups. SMs were required to carefully fill out the Hospital Anxiety Depression Scale (HADS) forms, and a correlation analysis was performed. ReHo was also utilized to appraise partial differences in spontaneous cerebrum function. Receiver operating characteristic (ROC) curves were created to partition ReHo values between patients with SM and the HCs.

**Results:**

ReHo values for the left cerebellum (LC), right fusiform gyrus (RFG), left inferior temporal gyrus (LITG), left rectus gyrus (LRG), right thalamus (RT), right caudate (RC), left insula (LI), and left thalamus (LT) of subjects with SM were notably higher than those of the HCs (*P* < 0.05). ReHo values of the right middle frontal gyrus (RMFG) in subjects with SM were decreased notably compared to the HCs (*P* < 0.05). ReHo values for the RMFG showed a negative correlation with the anxiety scores (ASs; *r* = −0.961, *P* < 0.001) and ReHo values for the RFG showed a positive correlation with the depression scores (DSs; *r* = 0.676, *P* = 0.016). The areas under the ROC curve were 1.000 (*P* < 0.001) for the RMFG, LC, LITG, LRG, RC, LI, and LT and 0.993 (*P* < 0.001) for the RFG and RT. The results from the ROC curve analysis indicated that changes in the ReHo values of some brain regions may help diagnose SM.

**Conclusion:**

Our research emphasized that patients with SM had lesions in synchronized neural activity in many encephalic areas. Our discoveries may provide beneficial information for exploring the neuromechanics of SM.

## Introduction

Adult obesity signifies body mass index (BMI) greater than or equal to 30, and severe obesity (SO) is defined as BMI greater than or equal to 35 (Hales et al., 2020). Obesity is now an epidemic disease and its complications include type 2 diabetes (Stein and Colditz, 2004) and increased risk for cardiovascular, musculoskeletal, and tumor diseases (D’Abbondanza et al., 2020). Apart from these widely known systemic diseases, SO gives rise to cognizance injures, and it is also a hazardous factor for vascular dementia. Some study have pointed out that the changes in cerebrum structure and function related to obesity may be the cause of cognitive and emotional dysfunctions in SO patients (Zeighami et al., 2021).

**FIGURE 1:**
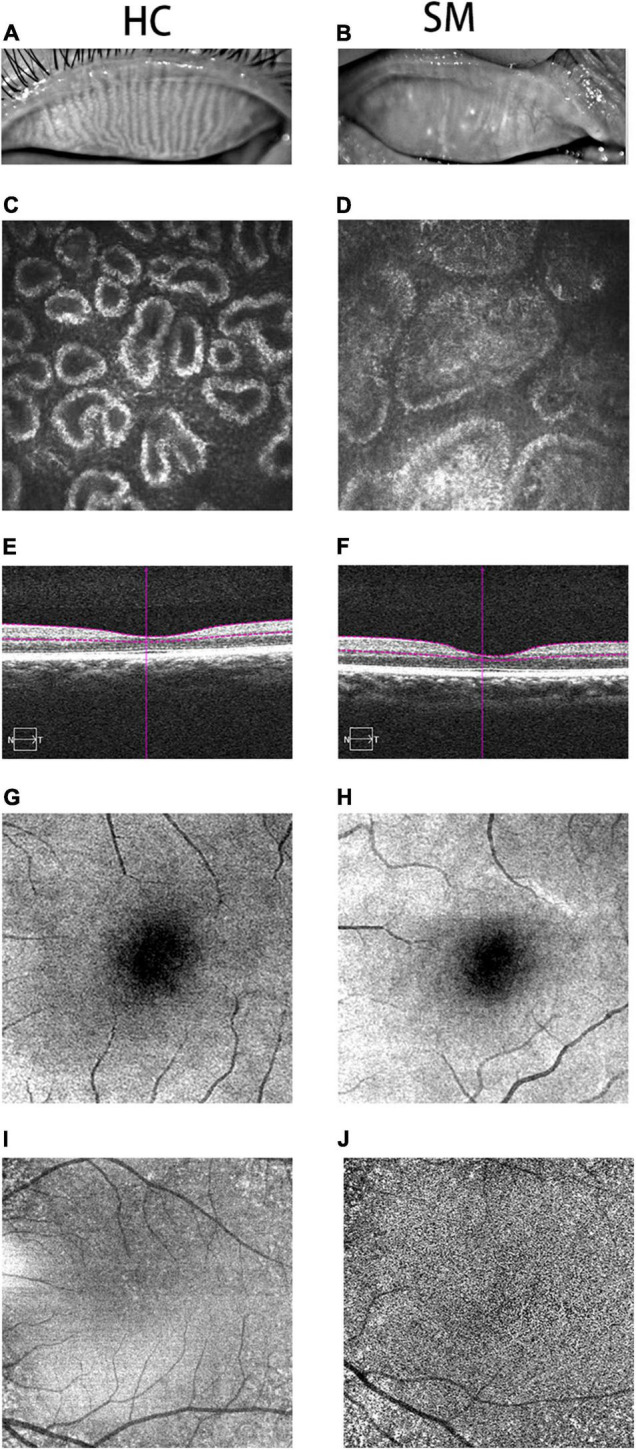
Typical pictures of HC and SM groups. We photographed the changes of MGs in HC group **(A)** and SM group **(B)**. We can obviously observed that the MG in HC group is clearer than that in SM group. At the same time, we took pictures of the two groups of MGs with corneal confocal microscope, and we could observe the typical differences between the two groups of MGs. The MG in SM group was obviously blocked, and it was widened at the same time **(D)**. Meanwhile, we could observe the normal MG in HC group **(C)**. Therefore, it could be speculated that severe obesity may lead to tarsal gland blockage and degeneration. According to the cross-sectional observation image of retinal thickness in the control group, we could observe that the choroidal thickness in the SM group **(F)** was significantly thinner than that in the HC group **(E)**. According to the fundus angiography images we obtained, we also observed that the capillaries in HC group **(G,I)** were more developed than those in SM group **(H,J)**, indicating that severe obesity may reduce the thickness of choroid and fundus blood supply, and may further cause fundus disease. HCs, healthy controls; SM, severe obesity and meibomian gland dysfunction; MG, meibomian gland.

The meibomian gland (MG) is a sebaceous gland in the eyelid. It can produce a tarsal plate and is the lipid component of the tear film. The plate gland is very crucial for delaying tear film evaporation, which can prevent dry eyes (Osae et al., 2019). The MG has the ability to reduce surface tension, which contributes to diffusion of the tear film on the ocular surface (Millar and Schuett, 2015). Due to the MG being able to produce lipids that make up the meibomian, and the blood and meibomian contour have attributes in common, some studies have reported that dyslipidemia, such as SO, can bring about meibomian gland dysfunction (MGD), including changes in the meibomian lipid component (Mudgil, 2014). An abnormality of the lipid component may lead to inflammation, which is an underlying cause of blepharitis and can eventually impact the regular excretory capabilities of the MG (Pietiläinen et al., 2007). This may generate degraded tear film quality and stabilization, eventually leading to ophthalmic surface inflammation in patients with MGD (Knop et al., 2011). A study showed that there was a strong positive correlation between dyslipidemia and MGD (Kuriakose and Braich, 2018). In the studied cases in our institution, we observed that all patients with SO had MGD. Due to the particularity of the anatomy of the MG, routine examination and diagnosis mainly rely on corneal confocal microscopy and fundus anatomy (Figure 1).

fMRI is a sophisticated non-invasive technique that is used to study the complexity of the brain–behavior relationship (García-García et al., 2019). Compared with traditional MRI technology, its main advantages include high dimensional resolution and the ability to disclose microstructure changes (Zhang et al., 2018). fMRI studies have reported differences in cerebrum activity levels between thin and obese people in different static state networks, including the default pattern network, significance network, and temporal lobe network (Val-Laillet et al., 2015). It is an essential tool for the study of cerebrum changes and the corresponding behavioral pathological basis in patients with SM.

The regional homogeneity (ReHo) method is a resting pattern functional magnetic resonance surveying method that has been used to assess lesions of the cerebrum (Guo et al., 2020). The ReHo method can measure the similarity of a time series of specific voxels and their nearest neighbors (Ding et al., 2020). These signals have previously been utilized to detect changes in nerve actions in sufferers of multifarious neural diseases, including Alzheimer’s disease, depressive disorder, schizophrenia, and attention deficit (Zhang Y. et al., 2019).

In this study, we utilized the ReHo method to study the cerebrum functions of patients with SM, to confirm lesions of cerebrum, and probe into the latent pathological mechanisms. To our knowledge, this is the first study on SM utilizing this approach.

## Materials and Methods

### Subjects

An aggregation of 12 patients with SM (4 men and 8 women) were enlisted from the Ophthalmology Department of the Nanchang University’s First Adjunctive Hospital. Indications for SM patients were as follows: (1) BMI ≥ 35; (2) male waistline ≥ 90 cm and female waistline ≥ 85 cm; (3) capable of being scanned with an MRI (no heart pacemaker or embedded metallic installations); and (4) no mental disturbance (major depression and/or anxiety disorders); (5) suffering from MGD. The elimination standard for SM included the following criteria: (1) SO caused by receiving massive hormones in a short period of time; (2) no painkillers before the fMRI scan; (3) history of diabetes; and (4) suffering from family inherited diseases.

Twelve (6 males and 6 females) subjects acted as healthy controls (HCs) and they were similarly aligned with SM sufferers in terms of age and gender. Every HC was conformed with the following standards: (1) 24 ≥ BMI ≥ 20; (2) could participate in an MRI scan; (3) head MRI showed normal brain parenchyma; (4) no history of MGD or other eye diseases; and (5) no mental illness.

### Ethical Approval and Consent to Participate

The study methods and protocols were approved by the Medical Ethics Committee of the First Affiliated Hospital of Nanchang University (Nanchang, China) and followed the principles of the Declaration of Helsinki. All subjects were notified of the objectives and content of the study and latent risks, and then provided written informed consent to participate.

### MRI Parameters

MRI scanning was operated on a 3-Tesla MR scanner (Trio, Siemens, Munich, Germany). High-distinguishability T1-weighted graphics were attained with a triaxial spoiled grads-duplicated sequence in an axial orientation: replication time = 1,900 ms, echo time = 2.26 ms, thickness = 1.0 mm, interval = 0.5 mm, collection matrix = 256 × 256, visual field = 250 × 250 mm, rollover angle = 9°. Definitely, 240 functional graphics (replication time = 2,000 ms, echo time = 30 ms, thickness = 4.0 mm, interval = 1.2 mm, collection matrix = 64 × 64, rollover angle = 90°, visual field = 220 × 220 mm, 30 axial sections with grads-duplicated echo-plane image formation pulse array) overlaying the entire cerebrum were attained.

### fMRI Data Analysis

All functional data were handled by a software percolator^1^ and statistical parameter graphing was operated with SPM8 (The MathWorks, Inc., Natick, MA, United States) and rs-fMRI DPARSFA^2^ software data conducting coadjutants. The primary procedures of pretreatment included slice timing, head motion rectification, exerting Friston six-head motion parameters to eliminate head motion influences, dimensional standardization with normal echo planar picture templates to achieve Neurology Montreal Institute (MNI) standards, and smoothening with a Gaussian kernel of 6 mm × 6 mm × 6 mm full-width at half-maximum (FW-HM). REST software (State Key Laboratory of Cognitive Neuroscience and Learning, Beijing Normal University, Beijing, China) was utilized to compute ReHo. The basal technique of assessment was to analyze Kendall consistency coefficients (KCC) of a given voxel and the adjacent voxel time series.

### Statistical Analysis

To probe the set differentiates in the ReHo values between patients with SM and the healthy, fMRI data were fitted with a general linear model (GLM) with the SPM9 toolkit. *P* < 0.05 was regarded as statistically crucial and rectified with random field (Gaussian random field) principle with minimum *z* > 2.3. Two-sample Student’s *t*-test was utilized for consecutive data for behavioral manifestations. All statistical analyses were accomplished with the IBM SPSS Statistics 20.0 software (IBM Corporation, Armonk, NY, United States).

### Brain–Behavior Correlation Analysis

In accordance with the ReHo computation consequences, some disparate cerebrum areas portrayed different semaphores between SM groups and HCs. For each area, the medial ReHo values was computed by meaning over all voxels. The connection between the average ReHo value and their clinical distinctions was computed utilizing the correlation analysis (*P* < 0.001 was deemed as statistically essential).

### Clinical Data Analysis

The accumulative clinical measured values, including the initial visual acuity, daily life scores and mini-mental state examinations were documented and analyzed in the research with standalone sample *t*-test (*P* < 0.05 as significantly different).

### Ocular Surface and Meibomian Gland Dysfunction Evaluations

We applied Keratograph ocular surface comprehensive analyzer to analyze the ocular surface. At the same time, we utilized MG evaluator and MG imaging technology to observe and evaluate the MG structure. According to the results of MG imaging, we scored and recorded the upper and lower eyelids of each patients’ eye, respectively. We scored according to the range of MG loss. Asian Dry Eye Association China Branch Score Standard: 0: no loss of MG; 1 point: the proportion of MG missing <1/3; 2 points: the proportion of missing MG is 1/3–2/3; 3 points: the proportion of MG loss is >2/3.1 or more is abnormal.

## Results

### Demographics and Visual Measurements

No remarkable differentiations in age (*P* = 0.365) and gender (*P* = 0.430) between SM subjects and HCs were identified. The distinctions observed between the two groups in initial visual acuity with binoculars, daily life scores, and mini-mental state examinations were statistically significant (*P* < 0.05; Table 1).

**TABLE 1:** Basic information of participants in the study.

Condition	SM	HC	*t*	*P*-value
Male/female	4/8	6/6	N/A	0.430
Age (years)	34.25 ± 7.07	31.67 ± 5.98	0.925	0.365
Weight (kg)	111.92 ± 13.33	66.08 ± 10.41	8.986	<0.01
Handedness	12R	12R	N/A	>0.99
Initial visual acuity-left eye (log Mar)	0.80 ± 0.16	0.58 ± 0.09	3.742	<0.01
Initial visual acuity-right eye (log Mar)	0.83 ± 0.22	0.62 ± 0.13	2.813	<0.05
Daily life score	90.92 ± 6.53	100.00 ± 0.00	4.617	<0.01
MMSE	21.42 ± 4.37	27.83 ± 2.41	4.266	<0.01

Independent t-tests comparing the two groups (p < 0:05 represented statistically significant differences). Data presented as mean ± SD. HC, healthy control; SM, severe obesity and meibomian gland dysfunction; MMSE, mini-mental state examination.

### ReHo Differences Between SM Patients and HCs

**FIGURE 2:**
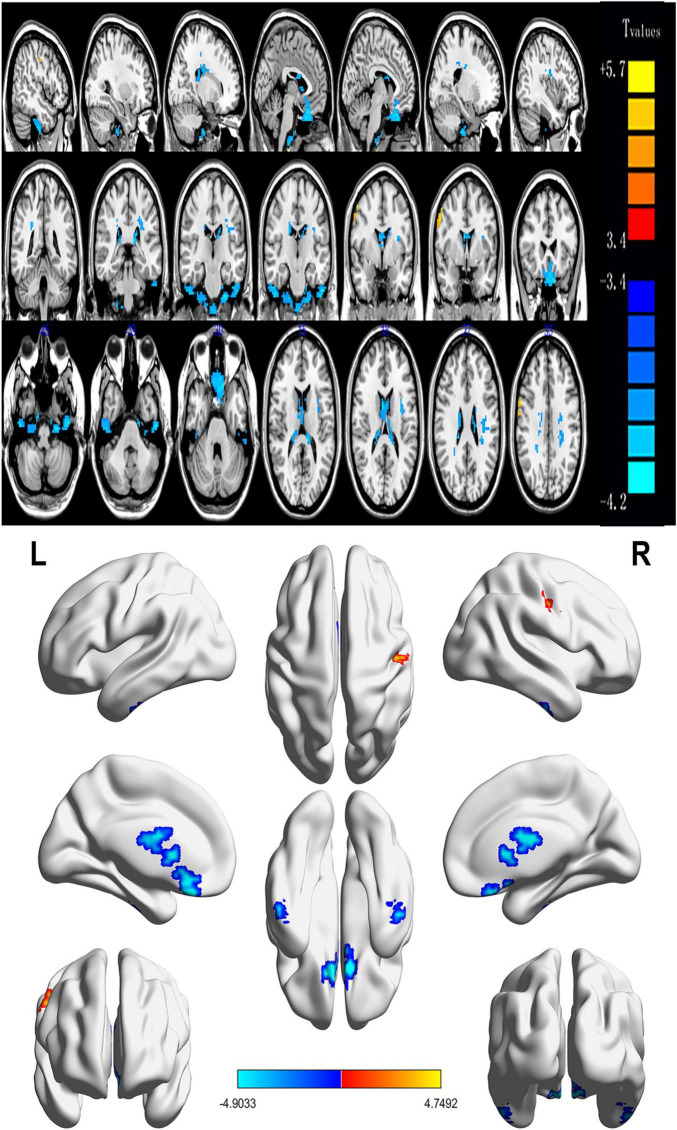
Spontaneous brain activity in patients with severe obesity and meibomian gland dysfunction. Red regions (left cerebellum, right fusiform gyrus, left inferior temporal gyrus, left rectus gyrus, right thalamus, right caudate, left insula, and left thalamus) indicate higher ReHo values, while blue regions (right middle frontal gyrus) represent lower ReHo values (*P* < 0.05; AlphaSim-corrected; cluster size, >40). ReHo, regional homogeneity; R, right; L, left.

**FIGURE 3:**
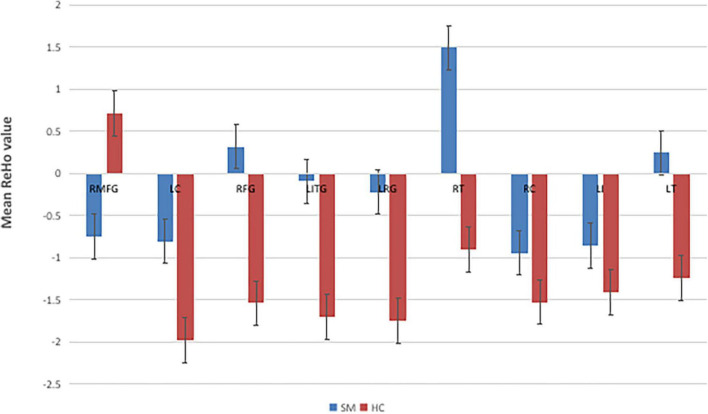
The mean single ReHo value between the SMs group and HCs. RMFG, right middle frontal gyrus; LC, left cerebellum; RFG, right fusiform gyrus; LITG, left inferior temporal gyrus; LRC, left rectus gyrus; RT, right thalamus; RC, right caudate; LI, left insula; LT, left thalamus; ReHo, regional homogeneity; HCs, healthy controls; SM, severe obesity and meibomian gland dysfunction.

Contrasted with HCs, SM sufferers manifested observably increased ReHo values in the left cerebellum, right fusiform gyrus, left inferior temporal gyrus, left rectus gyrus, right thalamus, right caudate, left insula, left thalamus, and reduced ReHo values in the right middle frontal gyrus (Figures 2, 3 and Table 2).

**TABLE 2:** Brain regions with significant differences in ReHo between the HC and SM groups.

ReHo	Brain areas	MNI coordinates					
		*X*	*Y*	*Z*	BA	Peak voxels	*t*-Value
HC > SO							
1	RMFG	54	0	51	6	85	7.28
HC < SO							
2	LC	−27	−21	−45		256	−9.61
3	RFG	51	−18	−45	20	108	−7.78
4	LITG	−54	−18	−39	20	107	−8.74
5	LRG	−12	24	−30	25	266	−9.38
6	RT	6	3	18		109	−8.87
7	RC	24	−42	33		109	−6.72
8	LI	−36	−21	27	13	97	−5.91
9	LT	−12	−33	15		80	−8.25

*For fMRI data, two-sample t-test was performed to examine the voxel-wise difference between the SM and HC groups using the REST toolbox. The statistical threshold was set at the voxel level with P < 0.05, FDR corrected, and cluster size >100 voxels for multiple comparison. These voxels were regarded as the regions of interest showing significant difference between the two groups. ReHo, regional homogeneity; HCs, healthy controls; SM, severe obesity and meibomian gland dysfunction; RMFG, right middle frontal gyrus; LC, left cerebellum; RFG, right fusiform gyrus; LITG, left inferior temporal gyrus; LRC, left rectus gyrus; RT, right thalamus; RC, right caudate; LI, left insula; LT, left thalamus.*

### Regional Homogeneity Values of Severe Obesity and Meibomian Gland Dysfunction Brain Regions and Hospital Anxiety Depression Scale Scores

**FIGURE 4:**
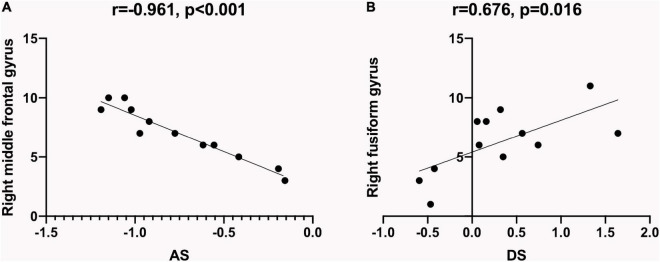
Relationship between ReHo values and emotional state. The ReHo values of specific brain regions in SM patients showed a correlation with HADS, which indicated that SM patients were more prone to be anxious and depressed. **(A)** ReHo values at the right middle frontal gyrus of the SM group showed a negative correlation with the AS (*r* = −0.961, *P* < 0.001). **(B)** ReHo values at the right fusiform gyrus of the SM group showed a positive correlation with the DS (*r* = 0.676, *P* = 0.016). ReHo, regional homogeneity; SM, severe obesity and meibomian gland dysfunction; AS, anxiety scores; DS, depression scores; HADS, hospital anxiety depression scale.

In the SM group, a negative correlation was found between the ReHo values at the right middle frontal gyrus (RMFG) and the anxiety scores (AS) (*r* = −0.961, *P* < 0.001; Figure 4A). ReHo values at the right fusiform gyrus (RFG) portrayed a positive correlation with the depression scores (DSs; *r* = 0.676, *P* = 0.016; Figure 4B).

### Receiver Operating Characteristic Curve

**FIGURE 5:**
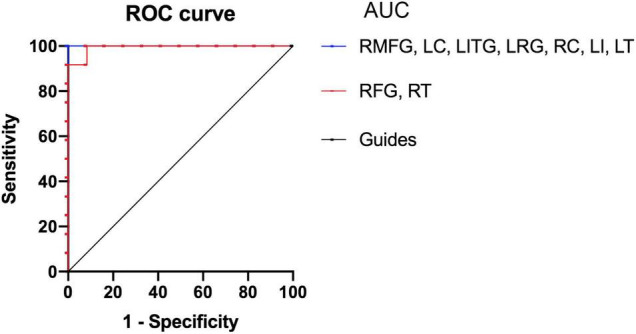
Receiver operating characteristic curve analysis of the mean ReHo values for altered brain regions. The areas under the ROC curve were 1.000 (*P* < 0.001; 95% CI: 1.000–1.000) for the RMFG, LC, LITG, LRG, RC, LI, LT; 0.993 (*P* < 0.001; 95% CI: 0.971–1.000) for the RFG, RT (SMs < HCs). HCs, healthy controls; SM, severe obesity and meibomian gland dysfunction; ReHo, regional homogeneity; ROC, receiver operating characteristic; AUC, area under curve; RMFG, right middle frontal gyrus; LC, left cerebellum; RFG, right fusiform gyrus; LITG, left inferior temporal gyrus; LRC, left rectus gyrus; RT, right thalamus; RC, right caudate; LI, left insula; LT, left thalamus.

There was an immense difference in the ReHo values between the SM group and the HCs. Therefore, we hypothesized that the ReHo values could be used to distinguish SM patients from the HCs. To test this hypothesis, we created an receiver operating characteristic (ROC) curve to investigate the medial ReHo values of disparate cerebrum areas. The area under curve (AUC) denoted the diagnosis rate. A value of 0.5–0.7 signified low accuracy, 0.7–0.9 signified medium accuracy, and >0.9 signified high accuracy. The areas below the ROC curve were 1.000 (*P* < 0.001; 95% CI: 1.000–1.000) for the RMFG, left cerebellum (LC), left inferior temporal gyrus (LITG), left rectus gyrus (LRG), right caudate (RC), left insula (LI), left thalamus (LT); 0.993 (*P* < 0.001; 95% CI: 0.971–1.000) for the RFG, and right thalamus (RT; Figure 5). Collectively, these results indicated that the ReHo values of disparate cerebrum areas might be used in the diagnosis of SM. In addition, ROC curves showed that the ReHo values of the RMFG, LC, LITG, LRG, RC, LI, and LT were more clinically relevant than the RFG and RT.

### Meibomian Gland Dysfunction Evaluation

We observed and evaluated the MG structure of SM group by MG imaging to determine the extent and degree of MG tissue loss. The results were: five people (one male, four female) one point; four people (one male, three female) two points; three people (two men, one woman) three points.

## Discussion

Nowadays, obesity has become an epidemic. According to research statistics, nearly 2.1 billion people in the world are overweight or obese (Smith and Smith, 2016). SO is often coupled with hyperlipidemia (D’Abbondanza et al., 2020), which is characterized by elevated blood lipid levels (causing atherosclerosis), such as cholesterol, triglycerides, and low-density lipoproteins, and decreased high-density lipoproteins (Butovich, 2008; Butovich, 2010; Green-Church et al., 2011). On the one hand, an abnormality of the lipid component will affect the structure of MG and make MG hypertrophy (Osae et al., 2020). On the other hand, it may lead to inflammation, which is an underlying cause of blepharitis and can eventually impact the regular excretory capabilities of the MG (Pietiläinen et al., 2007). This may generate degraded tear film quality and stabilization, eventually leading to ophthalmic surface inflammation in patients with MGD (Knop et al., 2011).

Obstructive sleep apnea hypopnea syndrome (OSAHS) is a common complication in obese patients. It is characterized by recurrent apnea and hypopnea during sleep, resulting in chronic intermittent hypoxia and hypercapnia (De Backer, 2013). Long term chronic intermittent hypoxia in patients with OSAHS will cause chronic inflammatory response and immune changes (Maniaci et al., 2021). Long term hypoxia and persistent inflammation lead to apoptosis of goblet cells and keratoconjunctival epithelial cells on the ocular surface and damage the ocular surface (Liu et al., 2022). In addition, this long-term intermittent hypoxia can not only directly damage the MG, but also indirectly cause MGD by causing the increase of matrix metalloproteinase, the decrease of elastic fibers and the relaxation of eyelids (Reins et al., 2018; Liu et al., 2022). The mechanism is that the MG cannot excrete normally due to the loss of eyelid support and extrusion, and MG obstruction develops into MGD (Pihlblad and Schaefer, 2013).

**FIGURE 6:**
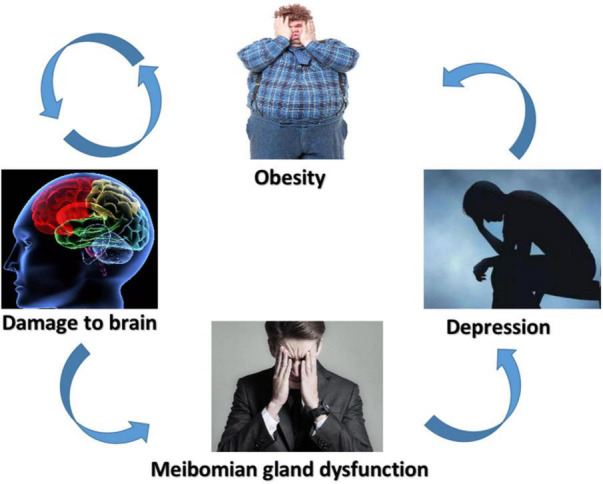
Schematic diagram of the relationship between SM, brain damage, MGD, and depression. SM, severe obesity and meibomian gland dysfunction; MGD, meibomian gland dysfunction.

To the best of our knowledge, this was the first investigation to assess the influences of SM on resting-state cerebrum motions utilizing the ReHo method. The ReHo technique has been used to study multiple systemic and neural diseases with success and it possesses massive potentiality for future studies (Table 3). In this research, we discovered that the SM group had reduced ReHo in the RFMG, and elevated ReHo in the LC, RFG, LITG, LRG, RT, RC, LI, and LT (Table 4). We also verified that the lesions in the internal connection patterns of SM patients’ brains were associated with a series of emotional and behavioral disorders including depression (Figure 6).

**TABLE 3:** Regional homogeneity method applied in systemic and neurogenic diseases.

	References	Disease
Systemic diseases	Liu et al., 2018	Systemic lupus erythematosus
	Zhang J. et al., 2019	Hypertension
	Zeighami et al., 2021	Hyperlipidemia
Neurogenic diseases	Xing et al., 2018	Primary Sjögren syndrome
	Ji et al., 2020	Schizophrenia
	Hu et al., 2020	Dementia
	Zhang et al., 2021	Diabetes
	Shen et al., 2020	Generalized anxiety disorder

*ReHo, regional homogeneity.*

**TABLE 4:** Brain regions alternations and its potential impact.

Brain regions	Experimental result	Brain function	Anticipated results
Right middle frontal gyrus	SMs < HCs	Continuous attention, emotional processing, sleep stability	Attention disorder, depression, primary insomnia
Left cerebellum	SMs > HCs	Emotional control, cognitive processing, motion control	Emotional and behavioral disorder
Right fusiform gyrus	SMs > HCs	Cognitive functions	Pure alexia, face blindness
Left inferior temporal gyrus	SMs > HCs	Mentality, visual word processing	Chronic schizophrenia, language barrier
Bilateral thalamus	SMs > HCs	Postural control, somatosensory and motor	Postural disorders, motor sensory dysfunction
Right caudate	SMs > HCs	Cognitive performance, memory, mental stability	Cognitive deficits, memory impairment
Left insula	SMs > HCs	Language expression, audiovisual perception, emotional control	Language barrier, audiovisual impairment, depression

*HCs, healthy controls; SM, severe obesity and meibomian gland dysfunction.*

The MFG is an area on the ventral side of the medial prefrontal cortex and it is thought to participate in emotional processing and automatic or implicit regulation of emotion (Jiang and Zuo, 2016). Moreover, it is responsible for many cognitive functions, such as top-down regulation in working memory, decision-making, attention processing, and emotional processing (Kupfer et al., 2012; Xu et al., 2020). In one study, researchers utilized amplitude of low-frequency fluctuations (ALFFs) measurements to study entire brain activity in patients with anxiety and depression. The outcomes showed the ALFF value in the MFG was decreased in anxious depressed patients. Therefore, the decrease in ALFF values in the MFG and its significant negative correlation with hysteresis factor scores indicated that the MFG is involved in the pathological process of anxious depression (Beevers et al., 2015). The decrease in MFG activity may also show the following comprehensive side effects: decreased cognitive function, reduced ability to change amygdala’s fear stimulus response by using emotion regulation strategies, thus weakening emotion regulation and increasing suicide possibilities (Japee et al., 2015). As is known, chronic attention deficit is a feature of many neurological diseases. fMRI has shown that the MFG is active in attention tasks and event analysis, indicating its importance in sustained attention (Zhao et al., 2020). In addition, interference from the right MFG can promote network associations with other areas to elevate persistent attentiveness. Another study emphasized that MFG acts an essential part of the kinematic network lesions involving attention conduction, especially the adjustment in persistent attention (van Heeringen et al., 2017). As one of the most common health problems, primary insomnia usually manifests as difficulty in starting and maintaining sleep (Neale et al., 2015). Research by Song et al. (2019) compared insomniacs with HCs and primary insomnia patients had reduced GM volume in several cerebrum regions, including the MFG. In another study, researchers evaluated the effects of 36 h of acute sleep deprivation on the functional connections between the PC and other areas of the brain. The results showed that 36 h after acute sleep deprivation, the connection between the right precuneus and the right MFG was markedly weakened (Morin et al., 2011). The authors suggested the right MFG acts as a significant factor in sustained attention, and damage to the right MFG may cause insomnia.

According to clinical observations and neuroanatomical research, the cerebellum is a structure closely connected with motor control as well as motor learning (Xie et al., 2020). The anatomic datum portrayed that the cerebellum had heterolateral connections with plentiful cortical regions of the bilateral hemicerebrums, including the motor area (D’Angelo et al., 2016; Li et al., 2020). One study have shown that sensory movement mainly activates the anterior lobule, and there are secondary manifestations in lobules VIIIa and VIIIB. In one study, healthy subjects were asked to reach out and grasp an object. During this process, the researchers detected the excitation of cerebellar lobules VI and VIIB (Bernard and Seidler, 2013). Recent studies have shown that, besides motor functions, the cerebellum also contributes to the stability of behavioral cognitive, emotional, and social functions (Bostan and Strick, 2018). These different functions can be activated because the cerebellum has obvious interconnections with different cortical areas. In addition, the prefrontal cortex-cerebellar circuit also exists and participates in the regulation of cognitive-emotional processes. Due to this versatility, cerebellar dysfunction and pathology can lead to cognitive and affective symptoms (Abdelgabar et al., 2019); for example, the skin pick-up disorder (SPD), which is a specific type of repetitive behavior of body focusing that can cause bodily harm. There have been reports that patients scratched their skin unconsciously until they noticed that they were scratching because of pain or bleeding (Van Overwalle et al., 2014). One study suggested that mood disorders were a core pathological mechanism of SPD. Before skin manipulation, the patient would feel a nervous or negative emotional state. By picking-up, the intensity of these disturbing states was at least temporarily reduced (Schmahmann and Sherman, 1998). Damage to the LC may cause negative emotions in SM patients, which may cause symptoms similar to the SPD.

Patients with pure aphasia show serious character recognition impairments. One study have shown that patients with dyslexia, after the damage to the RFG, are impaired not only in word recognition but also in recognition of numbers, objects, and even faces (Grant et al., 2012). Generally speaking, facial perception impairment refers to the inability to recognize previously seen faces (Roberts et al., 2013). This perception function is mediated by a well-defined and widely distributed hierarchical nervous system. The core of this system is composed of bilateral occipitotemporal regions of the striate extracorporeal visual cortex, the most significant of which is the RFG (Behrmann and Plaut, 2014). Therefore, damage to the RFG may lead to severe facial agnosia. In this research, we also discovered SM patients featured elevated ReHo values in the RFG region, suggesting that SM may be related to recognition impairment.

The inferior temporal gyrus is situated on the lateral and inferior surface of the temporal lobe (Susilo and Duchaine, 2013). Previous functional neuroimaging studies have shown that the inferior temporal gyrus is involved in a variety of cognitive processes, as well as the integration of vision and multimodal sensations (Cabeza and Nyberg, 2000; Conklin et al., 2002). These results indicated that sufferers with schizophrenia had a decline in gray matter volume in the bilateral inferior temporal gyrus (Herath et al., 2001). Furthermore, language fluency is one of the most commonly used neuropsychological measurement methods for language ability and executive function, requiring candidates to generate as many words as possible according to given category clues or letter clues within a preset time (Mesulam, 1998). Contrasted with the HCs, sufferers with left temporal region lesions spoke fewer words in the semantic fluency task (Onitsuka et al., 2004). Therefore, the lesion of the LITG in SM patients may cause mental instability and some language barriers.

The thalamus is not merely a simple relay function, it has a significant integration function, especially in vestibular processing. Peripheral organ fibers are mainly projected through the morphological specific thalamic nucleus, such as the external geniculate nucleus and medial geniculate nucleus of the optical system and the aural system, and then projected to the respective cortex or subcortical target area. This organization, together with the orderly corticothalamic feedback mechanism, turns the thalamus into a modulating character in sensorial processing (Shao et al., 2014). Therefore, for the vestibular system, the thalamus is a unique subcortical site of multi-sensory integration. These vestibular thalamic loops may construct functional passages that conform the signals of the vestibule with other signals in the thalamus (Luckhurst and Lloyd-Jones, 2001). Vestibular hemicrania is a central disorder of the features of aberrant perception (Sherman, 2007). fMRI of these sufferers during vestibular stimulation augmented the blood oxygen level-dependent signals in the thalamus were different from those of the healthy and migraine patients (Wijesinghe et al., 2015). Such observations indicated that the thalamus played an indispensable role in the production of vestibular perception, and the anomalies of the thalamus might be the cause of the distortions observed in some clinical conditions. The thalamic nucleus that receives the vestibular afferent is also concerned with handling messages from other sensorial systems. Thus, the thalamus may be regarded as an essential locus for sensory conformity. Studies have compared apoplexy patients with a certain extent of sensory loss with normal controls. The results showed that the interaction between vestibular and sensory messages relied on the functionality of the thalamus (Stolte et al., 2015). In addition, psychophysical studies have shown that the impairment of multisensory integration in patients suffering from Parkinson’s disease may be due to the lack of facilitation of the ascending cholinergic system, which leads to thalamic dysfunction (Russo et al., 2014). Moreover, a study of two Parkinson’s disease patients, after continuous cerebrum stimulation of the subthalamic nucleus, showed that subjective vertical changes in vision, a characteristic usually related to vestibular dysfunction, also indicated the interaction between vestibule and thalamus (Barra et al., 2010). The increased bilateral thalamic ReHo values associated with SM may cause migraine and motor-sensory dysfunction related to vestibular dysfunction.

The head of the caudate nucleus is part of the dorsolateral prefrontal circuit (DLPFC). Disruption of the DLPFC may lead to cognitive deficits in the prefrontal lobe and impaired memory (Müller et al., 2013). Lesions in the head of the caudate nucleus can lead to dysfunctional performance syndrome, attention deficits, and impaired short-term and long-term memory (Mike et al., 2009). Cognizant lesions in Parkinson’s disease patients impact multiple domains, such as attention, visual space, and memory (Leh et al., 2007). This may be due to the important function of the caudate nucleus as a message center, and dopaminergic degeneration of the caudate nucleus in Parkinson’s disease can lead to damage to the caudate nucleus (Apostolova et al., 2010). In one study, researchers found that the betweenness centrality of the RC nucleus of Parkinson’s disease patients was positively correlated with the Montreal cognitive assessment score (Bartels and Leenders, 2009). Through PET imaging, some have reported relevance between the RC nucleus and cognizant ability: they applied the Stroop test in sufferers with early Parkinson’s disease and found that the decline in the dopamine of the RC nucleus was associated with slow time processing and cognitive impairment (Bell et al., 2015; Wright et al., 2020). Therefore, damage to the RC of SM patients may cause cognitive and memory impairment.

The LI is located behind the frontal lobe, temporal lobe, and capillary cortex (Brück et al., 2001). Although overlying merely 2% of the cerebral cortex, the insula is like a multitudinous functional expressway involving a mass of cognitive and emotional courses. These include: visceral movement, visceral sensory function, body movement, motor correlation, eye movement, language system, and aural function, and it also covers cognitive domains, including physical awareness and emotion (Ko et al., 2017). The insula plays an important role in the language network. Guenot et al. (2004) carried out a meta-analysis of expressive language tasks and showed that the former island was the core part of activating language function. Recently, high-resolution fiber tract imaging studies have reported associations between the insula and the visually related regions of the parietal and temporal lobes in humans (Nieuwenhuys, 2012). In terms of visual function, the insula is considered to be a cross-modal cortical integrator that stimulates perception and visual awareness (Lu et al., 2016). Therefore, we speculated that SM and MGD might cause brain dysfunction (Table 4). Major depression is a persistent and debilitating emotional disorder characterized by negative emotions, difficulty sleeping, loss of appetite, and inattention (Chang et al., 2013). There has been evidence that the insula was an area related to emotional saliency and attention distribution during emotional tasks (Fletcher et al., 2015). Some data have shown that the insula was a pivotal brain structure for depression, emotional prominence, and mutual perception. Linear regression showed that abnormal LI thickness can significantly increase the risk of major depression (American Psychiatric Association, 2013). Therefore, the significant increase in the ReHo value of the LI may lead to speech and hearing impairment and depression.

In summary, evaluating the ReHo values of SM patients through fMRI is an efficient technique to probe into the connection between SM and lesions in cerebrum functions. We detected aberrant spontaneous activities in certain cerebrum regions of SM sufferers, which might be associated with the neurological mechanisms of SM. However, our research has a few limitations; for example, the sample size was too small, and all subjects were from the same institution. The general representation is, therefore, relatively poor. In addition, the scan time was too long for some participants, so their motions might have affected the results.

Also, our study lacked a detailed ocular surface assessment in SM patients. This may lead us to overlook the possible combination of obesity with other important eye diseases. Future research will expand the sample size, institutional sources, standardize the scanning procedures and mostly importantly, add detailed ocular surface assessments so as to obtain more representative and profound research results for SM.

## Data Availability Statement

The raw data supporting the conclusions of this article will be made available by the authors, without undue reservation.

## Ethics Statement

The study methods and protocols were approved by the Medical Ethics Committee of The First Affiliated Hospital of Nanchang University and followed the principles of the Declaration of Helsinki. All subjects were notified of the objectives and content of the study and latent risks, and then provided written informed consent to participate.

## Author Contributions

YL, S-XT, and Y-KW analyzed the data and drafted the manuscript. Y-KS, L-JZ, and MK assisted with data interpretation and figure composing. PY, Y-CP, and H-YS collected the data. YS conceived, designed, and directed the study, and revised and approved the manuscript. All authors contributed to the article and approved the submitted version.

## Conflict of Interest

The authors declare that the research was conducted in the absence of any commercial or financial relationships that could be construed as a potential conflict of interest.

## Publisher’s Note

All claims expressed in this article are solely those of the authors and do not necessarily represent those of their affiliated organizations, or those of the publisher, the editors and the reviewers. Any product that may be evaluated in this article, or claim that may be made by its manufacturer, is not guaranteed or endorsed by the publisher.

## Footnotes

1
www.MRIcro.com

2
http://rfmri.org/DPARSF
